# Platelet-Rich Plasma Improves the Wound Healing Potential of Mesenchymal Stem Cells through Paracrine and Metabolism Alterations

**DOI:** 10.1155/2019/1234263

**Published:** 2019-10-31

**Authors:** Barbara Hersant, Mounia Sid-Ahmed, Laura Braud, Maud Jourdan, Yasmine Baba-Amer, Jean-Paul Meningaud, Anne-Marie Rodriguez

**Affiliations:** ^1^Université Paris-Est, UMR-S955, UPEC, Créteil, France; ^2^INSERM U955, Créteil, France; ^3^AP-HP, Hôpital Henri Mondor-A. Chenevier, Service de Chirurgie Plastique et Maxillo-Faciale, Créteil, France

## Abstract

Chronic and acute nonhealing wounds represent a major public health problem, and replacement of cutaneous lesions by the newly regenerated skin is challenging. Mesenchymal stem cells (MSC) and platelet-rich plasma (PRP) were separately tested in the attempt to regenerate the lost skin. However, these treatments often remained inefficient to achieve complete wound healing. Additional studies suggested that PRP could be used in combination with MSC to improve the cell therapy efficacy for tissue repair. However, systematic studies related to the effects of PRP on MSC properties and their ability to rebuild skin barrier are lacking. We evaluated in a mouse exhibiting 4 full-thickness wounds, the skin repair ability of a treatment combining human adipose-derived MSC and human PRP by comparison to treatment with saline solution, PRP alone, or MSC alone. Wound healing in these animals was measured at day 3, day 7, and day 10. In addition, we examined in vitro and in vivo whether PRP alters in MSC their proangiogenic properties, their survival, and their proliferation. We showed that PRP improved the efficacy of engrafted MSC to replace lost skin in mice by accelerating the wound healing processes and ameliorating the elasticity of the newly regenerated skin. In addition, we found that PRP treatment stimulated *in vitro*, in a dose-dependent manner, the proangiogenic potential of MSC through enhanced secretion of soluble factors like VEGF and SDF-1. Moreover, PRP treatment ameliorated the survival and activated the proliferation of *in vitro* cultured MSC and that these effects were accompanied by an alteration of the MSC energetic metabolism including oxygen consumption rate and mitochondrial ATP production. Similar observations were found *in vivo* following combined administration of PRP and MSC into mouse wounds. In conclusion, our study strengthens that the use of PRP in combination with MSC might be a safe alternative to aid wound healing.

## 1. Introduction

Nonhealing wounds represent a major public health problem and a substantial economic burden for the healthcare system. They are found in many diseases including diabetes mellitus, ischemia, venous and pressure ulcers, or cancer or result from trauma, surgical act, or burn. The cost of wound care in the European Union accounts for 2-4% of the yearly healthcare budget and is expected to rise with the increase of elderly population aged over 65 years old and the growing prevalence of lifestyle diseases such as obesity and diabetes [1].

Despite the investment of significant healthcare resources in wound care, nonhealing wounds are associated with serious complications such as amputation for diabetic foot ulcers, disfigurement and scarring due to burns, and life-threatening functional handicap following degloving in the elders or sinus tracts (tunnels connecting abscesses) in hidradenitis suppurativa. Nonhealing wounds are also associated to cancer formation, especially squamous cell carcinoma, likely emerging from the repetitive tissue damage and the subsequent rapid cell proliferation. Therefore, there is a pressing need to develop novel strategies to replenish the skin loss resulting from acute, chronic, postinfection, and postinflammatory wounds, notably in elders and/or patient with significant history of diverse disorders.

Wound healing process requires a well-orchestrated sequence of events that include the coordination of many cells types like keratinocytes, fibroblasts, adipocytes, endothelial cells, macrophages, and platelets and the occurrence of several cellular changes in the wound site such as cell attraction, proliferation, and differentiation as well angiogenesis [2, 3].

By stimulating the body's own repair mechanisms, regenerative medicine offers the promise to regenerate nonhealing wounds through the development of strategies based on the use of cells, bioactive factors, and acellular skin substitutes [4]. Among these strategies, the administration of platelet-rich plasma (PRP) or mesenchymal stem cells has been intensively investigated to promote the regeneration of a broad range of soft and hard tissues including the skin [5]. PRP can be obtained in an autologous fashion, i.e., from the patient's blood through a centrifugation process leading to a plasma fraction with a platelet concentration higher than in circulating blood. A flurry of studies conducted in animal models or in human reported that PRP administration is beneficial for the treatment of chronic skin ulcer [6, 7], acute cutaneous wounds, burns [8], and plastic surgery [9, 10]. The therapeutic effects of PRP are mainly attributed to the release of growth factors by platelets upon their activation. These growth factors include platelet-derived growth factor (PDGF), epidermal growth factor (EGF), fibroblast growth factor (FGF), insulin-like growth factor (IGF-1, IGF2), and vascular endothelial growth factor (VEGF) that are known to favor tissue regeneration [11, 12]. Among these pleiotropic prohealing actions, platelet's growth factors have been found to stimulate the migration, the proliferation, and the differentiation of fibroblasts and endothelial cells to improve extracellular matrix secretion and angiogenesis, respectively, and to promote the chemotaxis of macrophages, monocytes, and polymorphonuclear cells to modulate inflammation [13]. In addition, the fibrin network generated following platelet activation contributes to tissue repair by providing a scaffold to the cells participating to the wound healing process at the site of injury [14]. However, despite the positive results obtained in preclinical studies, clinical trials using PRP have led to controversial outcomes [15–17].

On the other hand, the delivery of mesenchymal stem cells (MSC) constitutes a promising alternative to repair damaged tissues. Like PRP, MSC exert their prohealing effects primarily through the release of a broad range of soluble factors endowed with cytoprotective, proangiogenic, and anti-inflammatory properties including growth factors, cytokines, microvesicles, or exosomes [18]. However, in contrast to PRP, MSC adapt their secretome to the surrounding environment where they locate and their paracrine action can last several days after their engraftment [19]. MSC can be easily isolated from a broad range of tissues, but the most studied and frequently used in preclinical studies or clinical trials are MSC coming from bone marrow or adipose tissue.

According to their unique features, MSC have provoked great enthusiasm for their application in the treatment of nonhealing wounds. Indeed, several studies have revealed that MSC improve the neovascularization and the reepithelization of wounds, modulate the local inflammation, and mobilize resident stem cells to the site of injury [20]. In addition, their safety in cell therapies has been shown in studies for various diseases including cutaneous wounds [21].

Nevertheless, despite positive results obtained in animal models for tissue injury, clinical trials have revealed the limited ability of MSC in promoting skin healing [22]. These mitigated outcomes can be mainly explained by the poor survival of the engrafted MSC at the site of injury. This poor survival may be due to a low rate of proliferation after transplantation [23] or to massive cell death during the first days after transplantation [24, 25]. Thus, the survival of MSC following their engraftment is a critical parameter for the successful achievement of the cell therapy protocols. Many studies have attempted to optimize the efficacy of the MSC-based therapies by increasing their survival through genetic modification or pharmacological treatments [25–28]. However, although effective, these optimization methods are most of the time difficult to transpose to clinical applications.

As an alternative to these approaches, the use of PRP as clinical-grade adjuvant to enhance the therapeutic effectiveness of engrafted MSC has been suggested by several studies highlighting that PRP treatment improves the angiogenic potential of MSC both *in vitro* and *in vivo* [29, 30] and stimulates the proliferation of MSC *in vitro* [31, 32]. However, systematic studies on whether PRP alters the repair properties of engrafted MSC in skin wound healing are lacking. Herein, we investigated both *in vitro* and *in vivo* using a mouse model of full-thickness wound whether and how PRP improves the ability of MSC to regenerate damaged skin. In this attempt, we used human multipotent adipose-derived stem cells as an MSC model to conduct all the experiments [33].

## 2. Material and Methods

### 2.1. Platelet-Rich Plasma (PRP) Preparation

Platelet-rich plasma (PRP) was obtained through centrifugation of human blood collected from healthy volunteers according the RegenKit-BCT® procedures (RegenLab SA, Le Mont sur Lausanne, Switzerland). An average of 4.5 ml of PRP and 1.8 billion platelets were obtained from 8 ml of blood.

### 2.2. Cell Isolation and Culture

All the experiments were conducted with hMADS. As previously reported, these cells resemble to a cell line since they can be expanded more than 200 population doublings *in vitro* with apparent unchanged phenotype. HMADS cells were isolated from adipose tissues obtained from young donors after informed parental consent as previously reported [33].

HMADS cells were cultured in Dulbecco's modified Eagle's medium (DMEM), 1 g/l glucose, containing 10% heat inactivated fetal bovine serum (FBS) (Dominique Dutscher), 100 U/ml penicillin, 100 *μ*g/ml streptomycin, and 10 mM HEPES (Invitrogen). As described earlier [33], HMADS cells exhibited the following phenotype: CD44^+^, CD49b^+^, CD105^+^, CD90^+^, CD13^+^, Stro-1^−^, CD34^−^, CD15^−^, CD117^−^, Flk-1^−^, Gly-A^−^, CD133^−^, HLA-DR^−^, and HLA-I^low^.

Primary human umbilical vein endothelial cells (HUVEC) were purchased from PromoCell (Heidelberg, Germany). HUVEC cells were expanded on gelatin (2%)-coated dishes with the growth medium recommended and commercialized by the manufacturer (PromoCell). All cell types were maintained in a 5% CO_2_ atmosphere at 37°C.

For the *in vitro* studies, MSC were previously treated during 24 hours with FBS-free DMEM culture medium supplemented in heparin (20 U/ml) and containing 5%, 10%, or 20% PRP that correspond, respectively, to a platelet concentration of 20.10^6^/ml, 40.10^6^/ml, and 80.10^6^/ml medium.

### 2.3. Mouse Cutaneous Wound and Cell Injections

All experiments were performed according to institutional guidelines for animal care and were approved by the local ethics committee (COMETH approval # A-05194.02) and the French Ministry of Agriculture. We used the Galiano's murine healing model [34] because this model minimizes rodent wound contractions and therefore mimics wound healing processes occurring in humans including granulation tissue formation and reepithelialization. Six-week-old male mice C57BL/6JRj (Janvier Labs, Route du Genest, 53940 Le Genest-Saint-Isle, France) were anesthetized with isoflurane gas (Baxter, France) inhalation (2.5% in 500 ml/min of air), and surgeries were performed under standard sterile conditions. Four circulars, full-thickness 5 mm diameter cutaneous wounds were created on the back of each mouse, and sterile donut-shaped silicone splints with a diameter two times of the wound were fixed to the surrounding wound edge with an adhesive film (3M Ioban_TM2,_ 3M Science, St. Paul, MN, USA) and interrupted 6-0 silk thread sutures to prevent skin retraction. Immediately after the skin injuries, each wound was injected with 100 *μ*l of saline solution (HBSS) containing either 2 × 10^5^ HMADS cells alone or in combination with 20% PRP activated with 10% CaCl_2_. Control wounds were injected with either HBSS saline solution or CaCl_2_-treated 20% PRP alone.

The wounds were then covered with semiocclusive dressing (3M Tegaderm®, St. Paul, MN 55144-1000, USA). During all the experiments, mice daily received intraperitoneal injection of buprenorphine (0.1 mg/kg/day).

### 2.4. Wound Closure Analysis

Wound closures were blinded-quantified through the measure of the wound reepithelialization at day 3, day 7, and day 10 postsurgery, through a macroscopic analysis of the lesions on the dorsum of the mice. A disposable 15-centimeter medical paper wound measuring ruler was used to measure the wound size. The wound closure rate at day X postsurgery was calculated as the percentage of the wound area at day X compared with that postoperative day 0 as follows [1 − (wound area at day X postsurgery)/(wound area at day 0 postsurgery) × 100%].

### 2.5. Skin Elasticity Measurements

Skin elasticity and changes in skin viscoelasticity of the healed area were measured by cutometry (MPA 580, Courage & Khazaka electronic). A 2 mm diameter probe was used, and a constant suction of 450 mbar for 1 s followed by a relaxation time of 1 s was applied and repeated 3 times. The cutometer measures skin elasticity and viscoelastic proprieties *in vivo* based on the principle of suction/elongation using an optical measuring unit. Measurements were made on the right and left areas of the cheek at the same site for each assessment. The mechanical parameters *R*_2_, *R*_5_, and *R*_7_ were subsequently calculated. *R*_2_ refers to the gross elasticity of the skin, including viscous deformation. *R*_5_ refers to the net elasticity without viscous deformation and is represented by the immediate retraction/immediate distention ratio. *R*_7_ refers to recovery after deformation and corresponds to the portion of elasticity compared to the final distension. It is represented by the immediate retraction/final distension ratio.

### 2.6. Immunohistochemistry

Immunohistochemical measurement of angiogenesis was performed at day 3 and day 7 postsurgery. After animal sacrifice, regenerated wounds were harvested, fixed in 4% neutral buffered formalin for 48 h, dehydrated with a gradient alcohol series, cleared in xylene, and embedded in paraffin. Tissue sections (5 *μ*m) were deparaffinized, hydrated, and pretreated for antigen retrieval with citrate buffer. Sections were then incubated with a rat anti-mouse CD31 antibody (clone MEC 13.3, BD Pharmingen, 1 : 400) followed by exposure to Alexa Fluor 555 goat anti-rat IgG (Invitrogen, 1 : 500). Fluorescence was analyzed by conventional Zeiss Axioplan 2 Imaging microscopy.

Capillary density of healing area was determined by counting microvessels stained with isolectin B4 CD31 from at least 10 randomly selected fields/wound.

### 2.7. Real-Time PCR Assays

PCR assays were performed in samples from hMADS cells previously treated *in vitro* with different concentrations of PRP or following their engraftment into mouse wounds.

RNA from cultured cells and tissue were extracted by using TRIzol reagent (Invitrogen) or Fibrous Tissue Mini Kit (Qiagen), respectively. Reverse-transcribed was performed using the Superscript First-Strand Synthesis System (Invitrogen) and random primers. Quantitative RT-PCR reactions were performed in duplicate on a 7900 real-time PCR detection system (Applied Biosystems, Waltham, MA, USA) using Platinum SYBR Green qPCR SuperMix (Invitrogen) for transcriptional expression of human VEGF (forward 5′-AGAAGGAGGAGGGCAGAATCA-3′ and reverse 3′-CTCGATTGGATGGCAGTAGCT-5′), human SDF1 (forward 5′-GATTGTAGCCCGGCTGAAGA-3′ and reverse 3′-CCAGGTACTCCTGAATCCACTTTAG-5′), human ki67 (forward 5′-ACGTCGTGTCTCAAGATC-3′ and reverse 3′-CGGTACTGTCTTCTTTGAC-5′), and human 5-ATP synthase (forward 5′-GCCGGACTGGTCTCCAGAA-3′ and reverse 3′-ATGAGTGTTAGAGGCATGGAAGTTC-5′).

Human SFA3A1 (forward 5′-TGCAGGATAAGACGGAATCCAAA-3′ and reverse 5′-GTAGTAAGCCAGTGAGTTGGAATCTTTG-3′) and mouse GAPDH (forward 5′-GCTCTCTGCTCCTCCTGTTC-3′ and reverse 3′-ACTCCGACCTTCACCTTCC-5′) were used as reference genes.

### 2.8. Collection of Conditioned Media

HMAD cells seeded at 10^5^ cells/ml were exposed to various concentrations of PRP. Twenty four hours later, supernatants were collected, centrifuged at 4300 rpm for 5 min to remove cell debris and frozen.

### 2.9. ELISA Assays

Secretion of VEGF and SDF-1 by hMADS cells following PRP treatment was assessed using ELISA kits (Abcam) according to the manufacturer's instructions. Cytokine concentrations were calculated from calibration curves obtained from serial dilutions of respective recombinant standards. Cytokine concentrations of conditioned media containing PRP in the absence of hMADS cells were also measured and the values were, respectively, subtracted to the cytokine dosages obtained from conditioned media from MSC following exposure to the corresponding concentrations of PRP.

### 2.10. Angiogenesis Assays

Angiogenic effects of culture conditioned media were evaluated by 2D angiogenesis assay using human umbilical vein endothelial cells (HUVEC) as previously reported [35]. Briefly, HUVEC (PromoCell) were seeded at 25 × 10^3^ cells per well of 96 well-plate precoated with Matrigel (BD Pharmigen) and exposed to conditioned media from hMADS cells in the absence or after PRP treatment. As controls, conditioned media from PRP alone or hMADS cells alone were tested. After 24 hours, HUVEC junction number and tube length were quantified. For this, each well was photographed and images were analyzed using J 1.42q software (National Institutes of Health). The endothelial branch length and number following exposure to conditioned media from PRP-treated MSC were corrected by subtracting the values obtained with conditioned media from PRP alone.

### 2.11. Cell Migration Assay Using the Agarose Drop Method

HUVEC cells were resuspended in basal medium containing 0.3% low melting agarose at a density of 40 × 10^6^ cells/ml. Droplets (3 *μ*l) of the agarose cell suspension were seeded into 24-well plates coated with polyDL prior to be incubated with conditioned media from hMADS cells cultured during 24 hours in the absence or in the presence of 5%, 10%, and 20% PRP. Following a 24-hour exposure to conditioned media, the droplets were stained using the Diff-Quick kit (Medion Diagnostics AG, Dudingen, Switzerland) and pictures were taken with a microscope. The droplet area and total area (area of the droplet+area of migrating cells) were measured using the ImageJ software, and the cell migration index was determined by the ratio: total area/droplet area. The migration ratio obtained with conditioned media from PRP-treated hMADS cells was corrected by subtracting the migration area obtained with conditioned media from PRP alone.

### 2.12. Flow Cytometry Detection of Cell Survival following an H_2_O_2_ Insult

To induce oxidative stress-induced apoptosis, hMADS cells were exposed to FBS-free DMEM medium containing 600 *μ*M H_2_O_2_ for 2 h. After the stress, MSC were cultured during 24 hours in FBS-free DMEM culture medium in the absence or in the presence of 5%, 10%, or 20% PRP. The cells were then stained with Annexin V conjugated to phycoerythrin and 7AAD (BD Pharmingen) according to the manufacturer's protocol and analyzed by flow cytometry. The number of living hMADS cells was obtained by counting the double-negative stained cells and expressed as the percentage of the total cell count.

### 2.13. Seahorse Analysis

Real-time measurements of oxygen consumption rate (OCR), indicative of mitochondrial respiration, were determined in MSC following a 24-hour PRP treatment, using a Seahorse Bioscience XF24 Analyzer (Billerica, MA, USA). Cells were seeded at a density of 20,000 cells/well, and measurements were performed in FBS- and bicarbonate-free DMEM (pH 7.4) supplemented with 5.5 mM glucose, 1% GlutaMAX, and 1% pyruvate. Bioenergetic profiles of the cells were evaluated using the Agilent Seahorse XF Cell Mito Stress Test, with sequential additions of: 1 *μ*g/ml oligomycin (inhibitor of ATP synthase), 0.7 *μ*mol/l carbonyl cyanide 4-(trifluoromethoxy) phenylhydrazone (FCCP, uncoupling agent), and 1 *μ*M rotenone/antimycin A (ROT/AA, inhibitors of complex I and complex III of the respiratory chain, respectively). Baseline cellular OCR was initially measured, from which basal respiration was derived by subtracting nonmitochondrial respiration following addition of antimycin A/rotenone. ATP-linked respiration was calculated by subtracting the oligomycin rate from baseline cellular OCR. Proton leak respiration was calculated by subtracting nonmitochondrial respiration from the oligomycin rate. Maximal respiratory capacity was derived by subtracting nonmitochondrial respiration from the FCCP rate. Mitochondrial reserve capacity was calculated by subtracting basal respiration from maximal respiratory capacity. Coupling efficiency was determined by calculating the percentage of OCR immediately following the oligomycin treatment over the final baseline value.

### 2.14. ATP Assay

Intracellular ATP levels of hMADS cells following PRP treatments were measured using an ATPLiteTM Bioluminescence Assay Kit (PerkinElmer, France) according to manufacturer's instructions.

### 2.15. Statistical Analysis

Data analysis was performed using GraphPad Prism software version 6.0 (San Diego, CA). Data are expressed as mean ± SD, and statistical analysis one-way ANOVA combined with Bonferroni multiple comparison tests was applied. *p* values smaller than 0.05 were considered significant.

## 3. Results

### 3.1. PRP Treatment Improves the Healing Capacities of hMADS Cells following Their Engraftment into Mouse Wounds

To determine whether PRP treatment could improve the ability of MSC in regenerating the lost skin, hMADS cells and PRP were simultaneously delivered into mouse wounds. As controls, wounds were separately treated with either saline solution, PRP alone, or hMADS cells alone.

**Figure 1:**
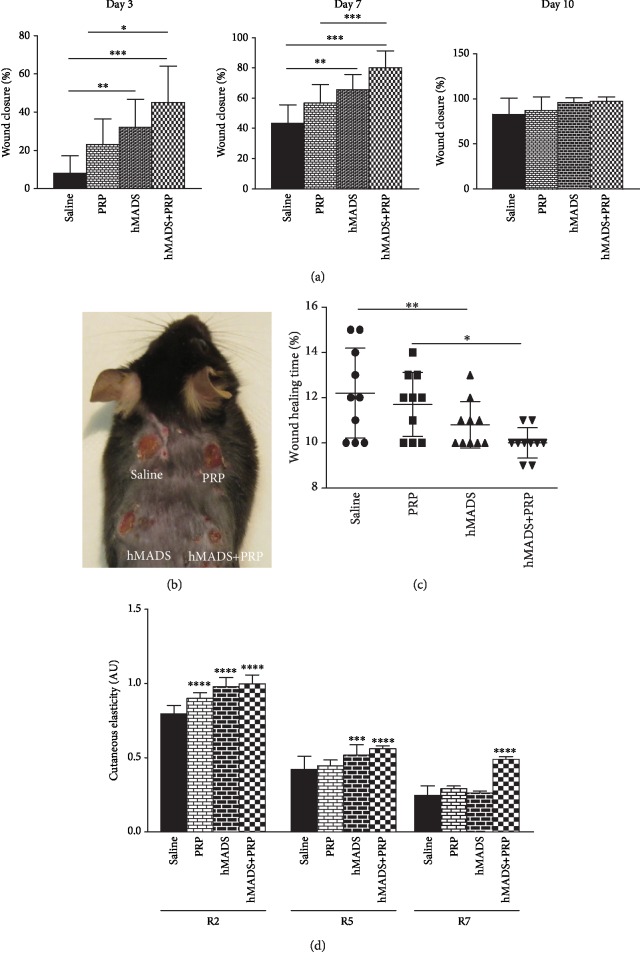
PRP treatment improves the healing potential of human MSC (hMADS cells) following their engraftment into mouse wounds. (a) Closure rates of wounds treated with saline solution, PRP, hMADS cells, or hMADS cells in combination with PRP at day 3, day 7, and day 10 following surgery. (b) Representative photograph of mouse back with skin wounds at day 3 following injury and treatment with either saline solution, PRP, hMADS cells, or hMADS cells in combination with PRP, respectively. (c) Wound healing times from mouse wounds treated with either saline solution, PRP, hMADS cells, or hMADS cells in combination with PRP, respectively. (d) Biomechanical parameters *R*_2_ (gross elasticity), *R*_5_ (net elasticity), *R*_6_ (viscoelasticity ratio), and *R*_7_ (biological elasticity) of the mouse healed wound following their treatment with either saline solution, PRP, hMADS cells, or hMADS cells in combination with PRP, respectively. (a–d) Results represent the mean ± SD obtained from *n* = 10 mice. ^∗^*p* < 0.05, ^∗∗^*p* < 0.01, ^∗∗∗^*p* < 0.001, ^∗∗∗∗^*p* < 0.0001.

The rate of the wound closure was determined by macroscopic analysis at day 3, day 7, and day 10 after injury (Figures 1(a) and 1(b)). For all time points, wound closure was found significantly higher in the group having received hMADS cells plus PRP than in the groups treated with either saline solution, PRP alone, or hMADS cells alone. At day 7 postsurgery, closure reached 80% for the wounds treated with both hMADS cells and with PRP, whereas 43% of closure was attained for control wounds treated with saline solution. However, at day 10 postinjury, complete closure was achieved for all groups (Figure 1(a)).

In agreement with macroscopic observations, we showed that the complete wound closure time corresponding to the total epimerization of the lesion was shorter when the wounds were treated with the hMADS cells in combination with PRP (10 days ± 0.6) by reference to the wounds administered with saline solution (12.2 days ± 1.9) (*p* < 0.01), PRP alone (11.7 days ±1.4), or the hMADS cells alone (10.8 ± 0.8) (Figure 1(c)). These results indicate that the use of PRP improves the therapeutic efficacy of engrafted MSC through acceleration of healing processes.

We then proceeded to the characterization of the elasticity of the new skin generated following the different treatments. For this purpose at day 15 postinjury, i.e., after complete wound healing, mechanical parameters including gross elasticity (*R*_2_), net elasticity (*R*_5_), and biological elasticity (*R*_7_) were measured. We found that *R*_2_ elasticity parameter was significantly increased in the skin generated following treatment with PRP, hMADS cells, or hMADS cells plus PRP by reference to that obtained after saline solution delivery (Figure 1(d)). In addition, the net elasticity *R*_5_ was also found to be significantly increased in healed wounds treated with hMADS cells or hMADS cells in combination with PRP comparing to the control (saline solution) treatment while no significant difference was observed following PRP treatment (Figure 1(d)).

In contrast, the *R*_7_ value that corresponds to the recovery from deformation was only found significantly improved in the healed wounds treated with hMADS cells plus PRP, by comparison to the control saline solution group (Figure 1(d)). These observations indicate that PRP maximizes MSC-based therapy not solely by significantly reducing the healing time but also by ameliorating the quality of the newly regenerated skin.

### 3.2. PRP Treatment Stimulates the Proangiogenic Properties of Engrafted hMADS Cells into Mouse Wounds

**Figure 2:**
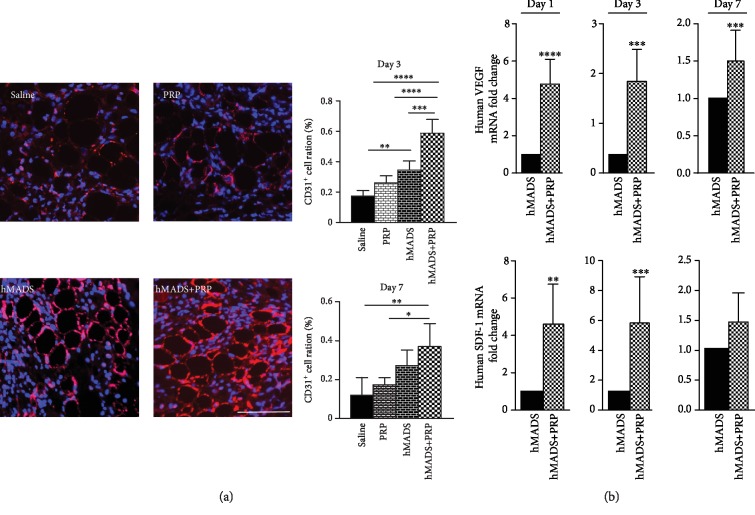
PRP treatment stimulates the proangiogenic properties of engrafted human MSC (hMADS cells) into mouse wounds. (a) Left panel: representative CD31 immunostaining of mouse wounds (red signal) at day 3 following treatment with saline solution, PRP, hMADS cells, or hMADS cells in combination with PRP, respectively. Nuclei were counterstained with Hoechst 33342 (blue signal). Scale bar, 50 *μ*m. Right panels: quantification of CD31-positive cells in mouse wounds at day 3 and day 7 following treatment with saline solution, PRP, hMADS cells, or hMADS cells in combination with PRP, respectively. (b) Relative human VEGF and human SDF-1 transcriptional expression in mouse wounds corresponding to the expression of hMADS cells engrafted in combination with PRP by reference to the expression in hMADS cells engrafted alone, at day 1, day 3, and day 7 postinjury. Results represent the mean ± SD obtained from at least *n* = 5 mice per group. ^∗^*p* < 0.05, ^∗∗^*p* < 0.01, ^∗∗∗^*p* < 0.001, ^∗∗∗∗^*p* < 0.0001.

In the attempt to determine the modalities by which PRP improves the therapeutic effectiveness of engrafted MSC, we started by analyzing the vascularization of the wounds following the delivery of saline solution, PRP alone, hMADS cells alone, or hMADS cells plus PRP. With this goal, immunohistochemistry with the endothelial CD31 marker was performed at day 3 and day 7 postinjury. In both time points, a significant higher number of endothelial cells was detected in the wounds treated with hMADS cells plus PRP compared to the other conditions (Figure 2(a)). In addition, we found that the transcriptional expression of human VEGF and human SDF1, two key factors involved in angiogenesis and migration processes, respectively, was significantly overexpressed in hMADS cells engrafted in combination with PRP, by comparison to the hMADS cells engrafted alone. The transcriptional upregulation of these genes was detected at day 1, day 3, and day 7 postinjury (Figure 2(b)).

Taken in concert, these findings suggest that PRP stimulates the ability of engrafted MSC to promote new vessel formation by stimulating their paracrine function and the release of proangiogenic soluble factors.

### 3.3. *In Vitro* PRP Exposure Enhances the Proangiogenic and Migratory Potential of Cultivated hMADS Cells

**Figure 3:**
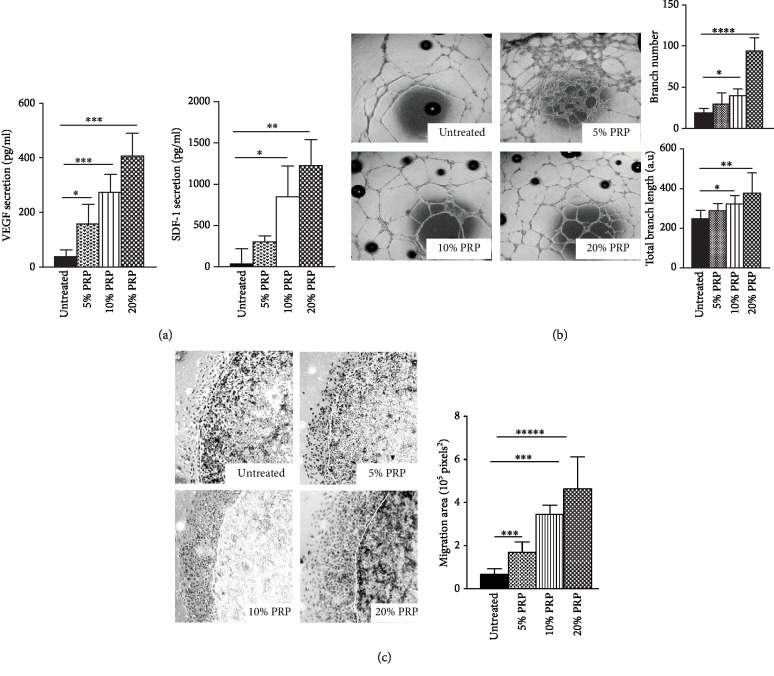
PRP treatment stimulates the proangiogenic properties of hMADS cells *in vitro*. (a) ELISA quantification of VEGF and SDF1 in conditioned media from hMADS cells after 24 hours of culture in the absence (untreated) or the presence of at 5%, 10%, or 20% PRP. (b) Left panel: representative images of capillary structures formed by endothelial HUVEC cells following a 48-hour exposure with conditioned media from untreated hMADS cells or hMADS cells treated with 5%, 10%, or 20% PRP. Right panel: capillary branch number and length after a 24-hour exposure of HUVEC cells to conditioned media from untreated hMADS cells or hMADS cells treated with 5%, 10%, or 20% PRP. (c) Left panel: representative images of the edge of agarose beads showing migration of HUVEC cells from agarose drops following a 24-hour exposure with the different conditioned media (hMADS cells grown in basal medium in the absence of PRP (untreated) or in the presence of 5%, 10%, or 20% PRP). White lines delineate the edge of the agarose droplets. Right panel: migration area quantification of HUVEC cells following a 24-hour exposure with the different conditioned media from hMADS cells in the absence of PRP (untreated) and 5%, 10%, or 20% PRP. Data represent mean ± SD of *n* = 5 independent experiments. ^∗∗∗^*p* < 0.001, ^∗∗∗∗^*p* < 0.0001.

To confirm the proangiogenic effect exerted by PRP on MSC *in vitro*, ELISA assays were performed to assess the concentration of VEGF and SDF-1 in conditioned media from hMADS cells following a 24-hour exposure to 5%, 10%, or 20% PRP (Figure 3(a)). These experiments showed that PRP significantly stimulated, in a dose-dependent fashion, the secretion of VEGF and SDF1 by hMADS cells (Figure 3(a)). In addition, the proangiogenic activity of conditioned media collected from hMADS cells following PRP treatment was evaluated in a HUVEC tube formation assay. We found that supernatants from hMADS cells previously treated with PRP, in comparison to supernatants from naïve hMADS cells, promoted higher angiogenesis of endothelial HUVEC cells. Indeed, the number and length of capillary branches formed by HUVEC cells were significantly enhanced in the presence of conditioned media from PRP-treated hMADS cells, with a PRP dose response, by comparison to conditioned media from untreated hMADS cells (Figure 3(b)). Finally, we showed that conditioned media from hMADS cells previously exposed to increased concentrations of PRP, induced in a dose-dependent fashion, a faster migration of HUVEC cells by reference to supernatants collected from untreated hMADS cells (Figure 3(c)).

### 3.4. PRP Treatment Improves the Survival of hMADS Cells and Stimulates Their Proliferation Both *In Vitro* and *In Vivo*

**Figure 4:**
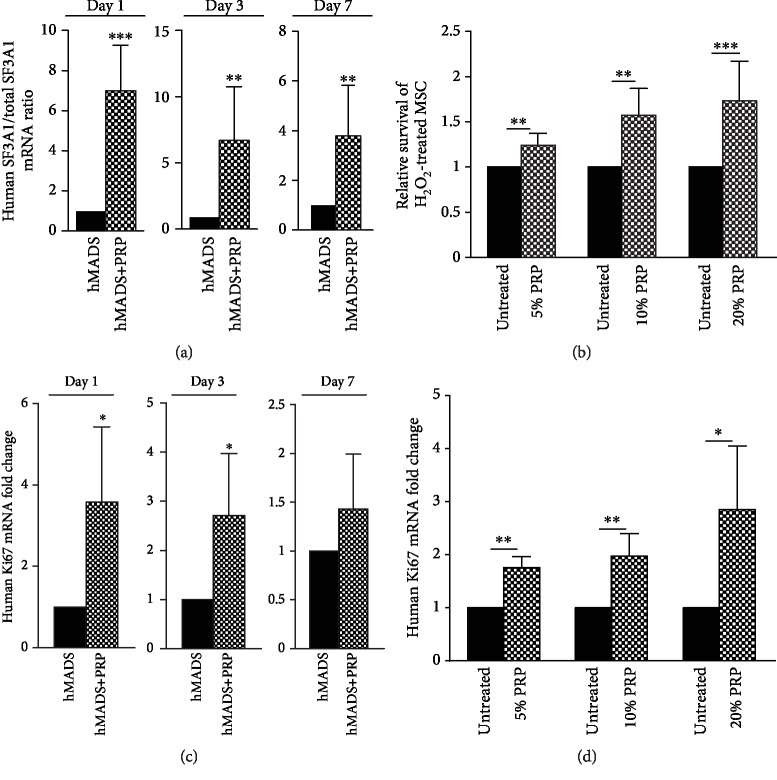
PRP treatment improves the survival and the proliferation of hMADS cells both *in vitro* and *in vivo*. (a) Relative survival of hMADS cells engrafted in combination with PRP in mouse wounds by reference to engrafted untreated hMADS cells, assessed by quantitative transcriptional expression of human SF3A1 gene, at day 1, day 3, and day 7. Data represent the mean ± SD of *n* = 6 mice per group independent experiments. ^∗∗^*p* < 0.01, ^∗∗∗^*p* < 0.001. (b) Relative survival of H_2_O_2_-injured hMADS cells following exposure to 5%, 10%, or 20% PRP by reference to PRP-untreated H_2_O_2_-injured cells (untreated) assessed by Annexin V/7ADD staining and flow cytometry analysis. Data represent the mean ± SD of *n* = 6 independent experiments. ^∗∗^*p* < 0.01, ^∗∗∗^*p* < 0.001, ^∗∗∗∗^*p* < 0.0001. (c) Relative human ki67 transcriptional expression in mouse wounds corresponding to the expression of hMADS cells engrafted in combination with PRP by reference to the expression of hMADS cells engrafted alone, at day 1, day 3, and day 7 postinjury. Results represent the mean ± SD obtained from at least  *n* = 5  mice per group.^∗^*p* < 0.05. (d) Relative ki67 transcriptional expression of hMADS cells following a 24-hour exposure to 5%, 10%, or 20% PRP by reference to hMADS cells grown in basal medium in the absence of PRP (untreated). Data represent the mean ± SD of *n* = 6 independent experiments. ^∗^*p* < 0.05, ^∗∗^*p* < 0.01.

Beyond the effect on angiogenesis, we wanted to determine whether PRP treatment might alter other properties of MSC accounting for their reparative impact such as survival and proliferation. We first assessed the survival rate of hMADS cells following their engraftment without or in combination with PRP. For this, we examined the level of human SF3A1 transcripts in grafted mouse wounds at day 1, day 3, and day 7 postinjury (Figure 4(a)). We showed that PRP treatment significantly increased the transcriptional expression of human SF3A1 from day 1 to day 7 postinjury suggesting that PRP improves the survival of engrafted hMADS cells. To assess whether the stronger engraftment of MSC was due to a cytoprotective effect of the PRP, we examined *in vitro* the viability of hMADS cells previously submitted to an injury in the absence or following PRP exposure. We choose to expose hMADS cells to an H_2_O_2_-induced oxidative stress as an injury model since oxidative stress occurs in mice in response to excisional wounds and because excessive reactive oxygen species (ROS) production is responsible of delayed or impaired skin repair processes [36]. By using Annexin V/7AAD staining and flow cytometry analysis, we demonstrated that PRP treatment conferred protection against apoptosis to hMADS cells compromised by a cytotoxic stress (Figure 4(b)). In addition, because the improved survival of hMADS cells found *in vivo* can also result to a greater proliferation, we investigated the transcriptional expression of the proliferative marker Ki67 in hMADS cells grafted in combination with PRP by reference to the expression of engrafted naïve MSC. We showed that PRP significantly increased the transcriptional level of Ki67 gene in grafted hMADS cells at day 1 and day 3 after wound injury while this overexpression persists but become not statistically significant at day 7 (Figure 4(c)). Consistent with the *in vivo* findings, PRP treatment was shown to activate, in a concentration-dependent manner, the ki67 transcriptional expression in cultivated hMADS cells (Figure 4(d)). Overall, these experiments indicate that PRP improves the graft maintenance/survival of MSC through cytoprotective and proliferative effects.

### 3.5. PRP Treatment Enhances the Survival of hMADS Cells through Likely Preservation of Their Energetic Metabolism

**Figure 5:**
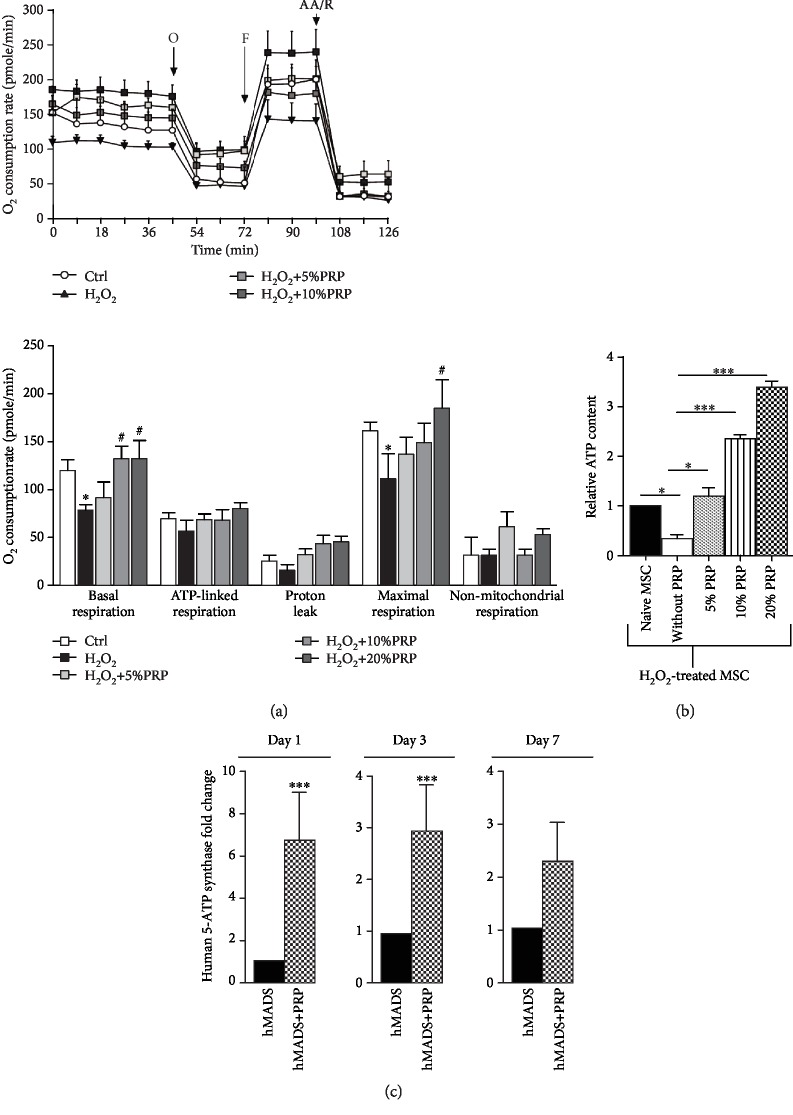
Effects of PRP on the energetic metabolism of hMADS cells. (a) Upper panel: representative graph showing oxygen consumption rate (OCR) measured by Seahorse XF analyser in hMADS cells in the absence or following a 24-hour treatment with 5%, 10%, or 20% PRP. A MitoStress assay was performed with a sequential addition of ATP synthase inhibitor oligomycin, electron chain uncoupler FCCP, and complex I and III inhibitors rotenone and antimycin A (R/AA). (b) Lower panel: mitochondrial respiration parameters including basal mitochondrial respiration (basal OCR measurement minus rotenone/antimycin A response) (basal), ATP-linked respiration (basal OCR measurement minus oligomycin response), proton leak (oligomycin response minus rotenone/antimycin A response), maximal respiration (FCCP response minus rotenone/antimycin A response), and nonmitochondrial respiration (rotenone/antimycin A response). Results obtained from *n* = 5 independent experiments are expressed as mean ± SEM; ^#^*p* < 0.05. (b) Relative ATP content of H_2_O_2_-injured hMADS cells in the absence (without PRP) or following treatment with 5%, 10%, or 20% PRP by reference to noninjured naïve hMADS cells. Data represent the mean ± SD of *n* = 5 independent experiments. ^∗^*p* < 0.05, ^∗∗∗^*p* < 0.001. (c) Relative human 5-ATP synthase transcriptional expression in mouse wounds corresponding to the expression of hMADS cells engrafted in combination with PRP by reference to the expression of hMADS cells engrafted alone, at day 1, day 3, and day 7 postinjury. Data represent the mean ± SD from *n* = 6 mice. ^∗∗∗^*p* < 0.001.

To determine whether the improved survival of MSC following PRP exposure involved metabolism alterations, we first performed MitoStress assays on H_2_O_2_-injured hMADS cells, in the absence or presence of PRP. We observed that H_2_O_2_-injured hMADS cells treated with PRP display a concentration-dependent increase in oxygen consumption rate (OCR) (Figure 5(a), upper panel), an increased ATP-linked respiration (or coupled respiration), and higher maximal respiration compared to untreated H_2_O_2_-injured MSC (Figure 5(a), lower panel). These results indicate that PRP treatment exerts cellular protective functions and counteracts metabolism dysfunctions induced by H_2_O_2_ injury in hMADS cells by restoring their mitochondrial respiration. Since ATP production is known to be critical to overcome metabolic stress due to cellular injury, we speculated that increased mitochondrial respiration in injured hMADS cells following PRP treatment might contribute to enhance their ATP production and subsequently their survival. To test this hypothesis, we measured the intracellular levels of ATP in H_2_O_2_-injured hMADS cells in the absence or following a 24-hour treatment with PRP (Figure 5(b)). As expected, we found that H_2_O_2_ injury decreased ATP production in hMADS cells by comparison to uninjured naïve MSC (Figure 5(b)). However, ATP drop was counteracted in H_2_O_2_-injured hMADS cells by PRP exposure. PRP significantly increased the ATP content in a dose-dependent fashion in damaged hMADS cells (Figure 5(b)). Interestingly, damaged hMADS cells treated with 10% or 20% of PRP contained higher ATP level than control uninjured cells (Figure 5(b)).

In agreement with these findings, the transcripts for the human 5-ATP synthase, a key enzyme involved in the mitochondrial ATP production, were shown overexpressed in hMADS cells when engrafted in combination with PRP by comparison to cells engrafted alone. This overexpression was detected at day 1, day 3, and day 7 postinjury, although this increase was not statistically significant at day 7 (Figure 5(c)). These results indicate that PRP stimulates the oxidative metabolism of damaged MSC and their ATP production, thus contributing to its cytoprotective effect.

## 4. Discussion

Acute or chronic wounds such as ulcers, diabetic wounds, or bedsores affect millions of people worldwide and represent a substantial economic burden for industrialized countries [37]. Since current treatments for wound care are still ineffective, the replacement of the lost skin remains a major challenge in the field of regenerative medicine. In this regard, the use of MSC represents a promising approach for the repair of damaged tissues or organs because (i) MSC can easily be isolated at clinical-grade standards from several tissues including bone marrow and adipose tissues [18], (ii) MSC secrete a number of soluble factors endowed with cytoprotective, trophic, and anti-inflammatory activities [18], and (iii) the use of MSC has been shown to be safe and feasible in the clinic (http://www.ClinicalTrials.gov). Although MSC are presently used in a wide array of clinical trials for several degenerative diseases, most of the completed trials have revealed modest efficacy of MSC in promoting regeneration of the damaged tissues including the skin [22, 38]. These disappointing outcomes are partly explained by a poor survival and retention of the MSC following their delivery into the damaged organs [23]. Therefore, the optimization of the clinical efficacy of MSC is needed. With this goal, several strategies have been developed to improve the viability, retention, and functionality of MSC based on genetic modifications, pharmacological preconditioning, or on the use of scaffolds or biomaterials [39–41]. However, most of these approaches are expensive and not easily translatable to humans.

As an alternative, we proposed in this study to determine whether PRP that is already used in clinic and whose separation from whole blood is not expensive could be employed as a source of growth factors, proteins, and enzymes to optimize the wound healing efficacy of MSC. Using a mouse model of full-thickness wounds and hMADS cells as a model of human MSC, we provided evidences that the combined administration of PRP and MSC is more efficient in promoting skin regeneration than the delivery of either PRP or MSC alone. Our findings are in agreement with previous reports showing that PRP enhances the repair potential of MSC following their administration into acute or diabetic wounds in pig or rat models, respectively [30, 42, 43]. However, whether the therapeutic effect of the combined treatment of PRP and MSC results to the sum of the separate effects of the two biological compounds or rather to a synergistic action between them has never been formally addressed. Our study highlights this issue by providing evidences that PRP exposure modulates the behavior of MSC following their engraftment into mouse wounds. In particular, we showed that PRP stimulated in engrafted MSC, their proangiogenic potential, their proliferation, and their survival. These *in vivo* observations were also confirmed through *in vitro* experiments.

First of all, we showed that the administration of PRP in combination with MSC promoted higher vascularization of the wounds than the delivery of MSC or PRP alone. Similar findings have been previously reported following the combined delivery of MSC and PRP in mouse ischemic hindlimb [29] or in pig and rat full-thickness wounds [30, 42]. Concerning the mechanisms underlying the improvement of angiogenesis in the treated wounds, our *in vivo* experiments clearly showed that PRP stimulated the transcriptional expression of proangiogenic factors in engrafted MSC. These results suggest that PRP stimulates the proangiogenic potential of engrafted MSC through activation of their secretome. Although not formally demonstrated by our *in vivo* experiments, this hypothesis is supported by our *vitro* experiments showing that MSC following PRP treatment exhibit a greater ability to promote vessel formation and endothelial cell migration and that these phenomena are accompanied by an increased secretion of VEGF and SDF-1 by cultured MSC.

Beyond the impact on MSC-mediated angiogenesis, our study reveals that PRP also enhances the therapeutic efficacy of engrafted MSC by favoring their retention/persistence to the wound site. This issue is of critical importance because the poor survival of engrafted MSCs is one major hurdle that compromises the effectiveness of cell therapy protocols. The greater number of MSC that persist to the site of injury following combined PRP delivery likely reflects a stimulatory action of PRP on the viability and proliferation of grafted cells. Our *in vivo* observations arguing these possibilities are strengthened by our *in vitro* experiments showing that PRP protects against apoptosis MSC previously submitted to an oxidative stress and stimulates their proliferation. To trigger cell death, we choose to expose hMADS cells to an oxidative stress-induced H_2_O_2_ treatment rather than an ischemic insult because hypoxia preconditioning in 5% O_2_ and 1% O_2_ does not decrease their survival. In contrast, our unpublished observations suggest that hypoxia preconditioning stimulates the repair properties of hMADS cells. Although similar *in vitro* findings related to the impact of PRP on MSC survival and proliferation have been previously reported [44–46], our study provides the first *in vivo* evidence that PRP affects these two features in MSC following their engrafted into mouse wounds.

Finally, one of the most exciting insights provided by our study concerns the mechanisms by which PRP exerts its cytoprotective function on MSC. Our results strongly suggest that this process involves alteration of the MSC metabolism to ensure a better adaptation/survival following their engraftment into the hostile/ischemic environment encountered in wounds. In particular, we reported for the first time that *in vitro* PRP exposure leads to an increased oxygen consumption and ATP-linked respiration in MSC previously injured by an H_2_O_2_ oxidative stress. As a consequence, this increased mitochondrial respiration leads to an enhanced ATP production. This higher intracellular ATP content should explain, at least in part, why PRP improves the viability of stressed MSC. Although further experiments are clearly needed to finely determine how and whether PRP modifies the metabolism of MSC, the fact that MSC administered concomitantly with PRP exhibit increased transcriptional expression of ATP synthase, a key enzyme involved in mitochondrial ATP production, suggests that similar process occurs *in vivo* and that PRP improves the energetic metabolism of infused MSC, thus leading to a better engraftment and functionality.

In conclusion, our study supports that PRP can be used as adjuvant to boost the wound healing efficacy of MSC by improving their proangiogenic, their survival, and their proliferative potential. In addition, our study reveals that the prostimulatory effects of PRP on MSC involve metabolism alterations leading to a better adaptation of engrafted MSC to their local environment. Future prospects in this field might elucidate the mechanisms by which PRP affects the regenerative properties of MSC in order to develop more efficient strategies to treat nonhealing wounds and other degenerative diseases.

## Data Availability

The data used to support the findings of this study are available from the corresponding author upon request.
